# Cutaneous manifestations of inflammatory bowel disease: basic characteristics, therapy, and potential pathophysiological associations

**DOI:** 10.3389/fimmu.2023.1234535

**Published:** 2023-10-26

**Authors:** Ronghua He, Subei Zhao, Mingyu Cui, Yanhao Chen, Jinrong Ma, Jintao Li, Xiaodong Wang

**Affiliations:** ^1^ Department of Gastroenterology, The Second Hospital of Jilin University, Changchun, Jilin, China; ^2^ Department of Endocrinology, The First Affiliated Hospital of Chongqing Medical University, Chongqing, China

**Keywords:** inflammatory bowel disease, Crohn’s disease, ulcerative colitis, extraintestinal manifestations (EIMs), cutaneous manifestations, pathophysiological associations

## Abstract

Inflammatory bowel disease (IBD) is a chronic inflammatory disease typically involving the gastrointestinal tract but not limited to it. IBD can be subdivided into Crohn’s disease (CD) and ulcerative colitis (UC). Extraintestinal manifestations (EIMs) are observed in up to 47% of patients with IBD, with the most frequent reports of cutaneous manifestations. Among these, pyoderma gangrenosum (PG) and erythema nodosum (EN) are the two most common skin manifestations in IBD, and both are immune-related inflammatory skin diseases. The presence of cutaneous EIMs may either be concordant with intestinal disease activity or have an independent course. Despite some progress in research on EIMs, for instance, ectopic expression of gut-specific mucosal address cell adhesion molecule-1 (MAdCAM-1) and chemokine CCL25 on the vascular endothelium of the portal tract have been demonstrated in IBD-related primary sclerosing cholangitis (PSC), little is understood about the potential pathophysiological associations between IBD and cutaneous EIMs. Whether cutaneous EIMs are inflammatory events with a commonly shared genetic background or environmental risk factors with IBD but independent of IBD or are the result of an extraintestinal extension of intestinal inflammation, remains unclear. The review aims to provide an overview of the two most representative cutaneous manifestations of IBD, describe IBD’s epidemiology, clinical characteristics, and histology, and discuss the immunopathophysiology and existing treatment strategies with biologic agents, with a focus on the potential pathophysiological associations between IBD and cutaneous EIMs.

## 1 Introduction

IBD is an immune-mediated chronic inflammatory disease mainly involving the gastrointestinal tract. It is caused by the interaction of multiple factors, including genetic, environmental, immune, epithelial, and microbial, making it complex (1). The global incidence and prevalence of IBD vary widely by geographic region, ranging from 0.1 to 58 cases per 100,000 person-years and 0.9 to 505 cases per 100,000 population, respectively; the highest rates have been reported in North America and Europe (2). In recent years the incidence has also increased rapidly in many newly industrializing countries (3). IBD’s peak usually occurs between the ages of 20–40 years but a moderate second peak occurs after the age of 60 years (4). In Asia, IBD is more common in males, while in the Western world, patients with IBD are predominantly females (5). Depending on the location and depth of intestinal involvement, IBD is divided into two subtypes, CD and UC (6).

Although IBD is a chronic inflammation of the gut, the manifestations of CD and UC are not limited to the gastrointestinal tract. EIMs are frequently observed in patients with IBD, with a reported frequency of 6%–47% (7). The skin, musculoskeletal, joints, eyes, and hepatobiliary tract are the organs most affected by the EIMs (8, 9). Of these, cutaneous EIMs are the most frequently reported, with an incidence of more than 17% (7). According to the different pathophysiological mechanisms, cutaneous EIMs are divided into the following four categories: (1) specific skin manifestations with the same histological features as IBD, such as metastatic CD; (2) reactive skin manifestations with similar pathophysiological mechanisms to IBD, such as PG, Sweet’s syndrome, EN, and aphthous ulcer; (3) IBD-related skin diseases, such as psoriasis, hidradenitis suppurativa, and atopic dermatitis, and (4) IBD-treatment-induced skin lesions, such as anti-tumor necrosis factor (TNF)-α-induced skin eruptions (10). A clinical cohort study by Hung et al. showed that patients with IBD are at different degrees of risk of having certain skin lesions at or after the diagnosis of IBD (11). PG and EN are the two most common skin manifestations in IBD (12) and both are immune-mediated inflammatory skin diseases. In addition to the association between cutaneous EIMs and intestinal disease, various cutaneous EIMs are linked. For example, PG and EN are related, while EN is also associated with aphthous ulcers (13). Based on the correlation between different cutaneous EIMs and between cutaneous EIMs and IBD, along with the recently confirmed efficacy of biologic agents for IBD and cutaneous EIMs, the proposed hypothesis is that there may be a potential pathogenic association between these conditions. This potential pathogenic association is not fully understood but it is speculated that damage to the gastrointestinal mucosa in patients with IBD may trigger excessive immune responses in the skin through multiple pathways. In this review, we provide an overview of the two most representative cutaneous manifestations of IBD; describe its epidemiology, clinical characteristics, and histology, and discuss the immunopathophysiology and applicable treatment options with biologic agents, with a focus on the potential pathophysiological associations between IBD and cutaneous EIMs.

## 2 Immunopathophysiology of IBD

**Figure 1:**
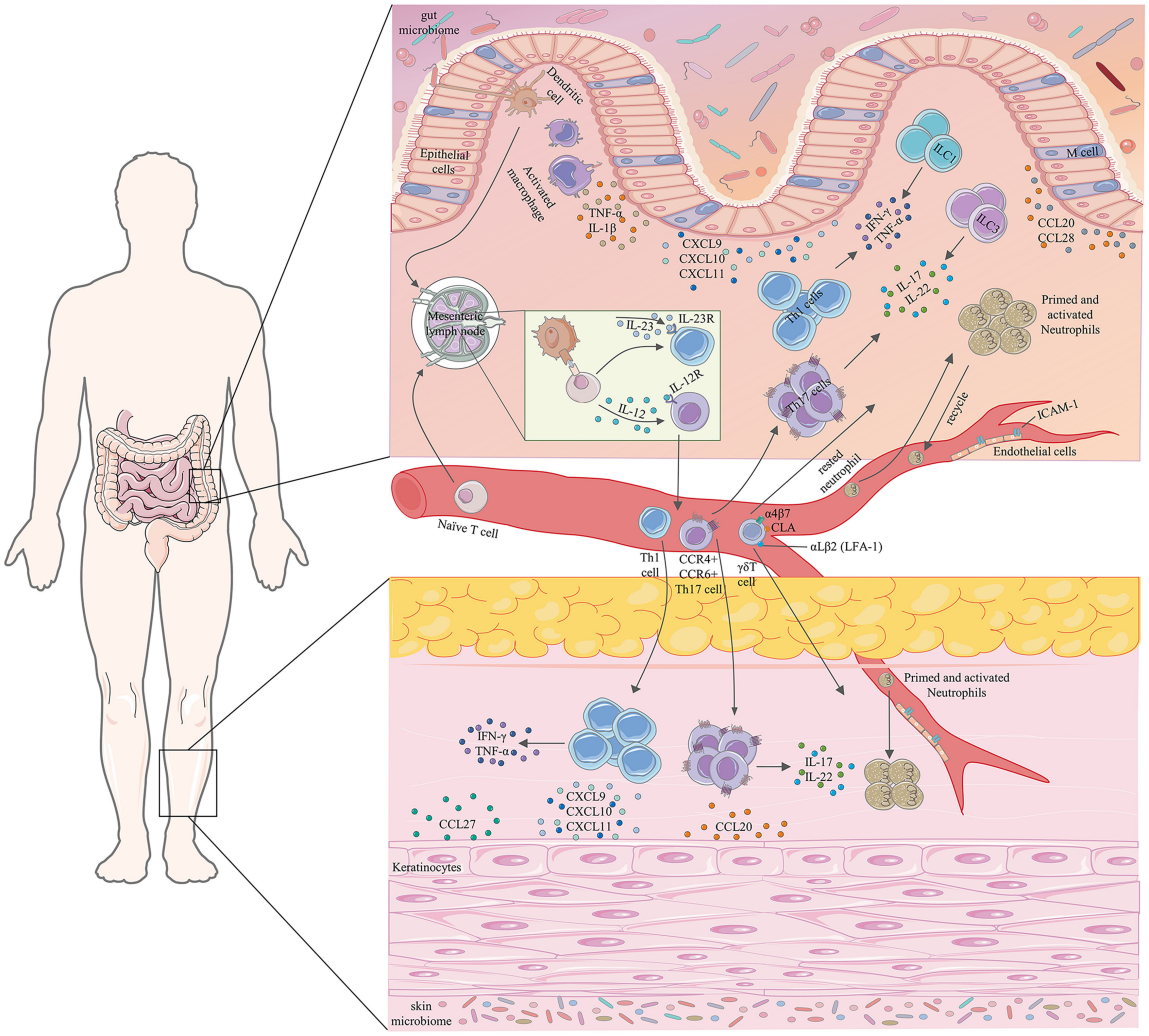
Partial pathophysiological associations between IBD and cutaneous EIMs. TNF-α, tumor necrosis factor-α; IFN-γ, interferon-γ; IL, interleukin; CXCL, C-X-C motif chemokine ligand; ILCs, innate lymphoid cells; CLA, cutaneous lymphocyte-associated antigen; LFA-1, lymphocyte function-associated antigen-1; ICAM-1, intercellular adhesion molecule-1; Th1, type 1 helper T; Th17, type 17 helper T.

Genetic, environmental, epithelial, microbial, and immune factors are involved in the occurrence and development of IBD. In IBD, gut microbiota dysregulation is associated with mucus layer disruption, epithelial cell tight junction dysregulation, defective Paneth cell number and function, and increased gut permeability, leading to increased gut microbial exposure (1). Antigen-presenting cells (APCs) continually recognize and sample these exposed microorganisms and produce abundant pro-inflammatory cytokines such as TNF-α, interleukin (IL)-1, IL-6, IL-12, and IL-23, and then migrate to mesenteric lymph nodes (MLN) or gut-associated lymphoid tissue (GALT), where they present antigens to naïve CD4+T cells. Under the influence of these pro-inflammatory cytokines, conserved pathogenic structures, or danger signals released by damaged cells, naïve CD4+T cells undergo proliferation and differentiation into effector T cell subsets comprising type 1 helper T (Th1) and type 17 helper T (Th17) cells (1). These primed and activated effector T cell subsets migrate out of the MLN or GALT and move into the bloodstream (14, 15). Under the interaction of chemokines with chemokine receptors and integrins with cell adhesion molecules, they migrate from the blood along chemotactic gradients to the lamina propria of the intestinal mucosa (14). Effector T cell subsets homing to the gut adapt the composition of their surface molecules (e.g., upregulation of integrin αEβ7) in response to the inflammatory circumstances, thereby allowing them to reside in the tissue (16). These effector T cell subsets residing in the intestinal mucosal lamina propria produce pro-inflammatory cytokines associated with prolonged intestinal mucosal inflammation and tissue damage (1, 17, 18) (**Figure 1**).

### 2.1 Chemokines in IBD

Chemokines and adhesion molecules are crucial components of our immune system and regulate the migration and localization of immune cells in tissues. Extensive analysis of various immune cells in the innate and adaptive immune systems has revealed their different migratory capacities, which are reflected in the unique chemokine receptors expressed by the various immune cells themselves, that mediate the migration of immune cells in the direction of increasing chemokine concentration. In addition to mediating the migration of immune cells themselves, chemokine receptors can help distinguish between different cell subsets, such as Th1 cells (CXCR3+) and Th17 cells (CCR4+CCR6+) (19, 20). The ligands of CXCR3 include CXCL9 (a monokine induced by interferon (IFN)-γ), CXCL10 (IFN-induced protein-10), and CXCL11 (IFN-inducible T-cell alpha chemoattractant), which are produced by human intestinal epithelial cells, are secreted in large quantities in an inflammatory environment rich in IFN-γ and can recruit CXCR3+ cells to the inflammatory site (21, 22). Overexpression of chemokine pairs CXCR3-CXCL9, CXCL10, and CXCL11 in human IBD has been demonstrated (23, 24). They are tightly associated with Th1 cell responses (19, 25) and increased secretion of pro-inflammatory cytokine by monocytes, especially with IFN-γ, to synergistically induce the secretion of IL-12 and IL-23 (26). CCL20 is also a chemokine secreted by human intestinal epithelial cells, which can chemotactically attract Th17 cells expressing CCR6 and is closely associated with inflammation and homeostasis (27). The expression of CCR6 is a common feature of IL-17-secreting cells, such as Th17 cells and γδT cells (20, 28). Multiple pro-inflammatory cytokines (e.g., TNF-α) induce CCL20 expression, and high upregulation of the CCR6-CCL20 axis has been demonstrated in the intestinal mucosa of patients with IBD (27, 29, 30). Meanwhile, Soler et al. found that Th lymphocytes expressing CCR4 can be mediated to home to the skin (31). CCR9-CCL25 plays an indispensable role in the recruitment of intestinal immune cells. CCL25 is the only receptor for CCR9, which is expressed not only by small intestinal epithelial cells under homeostatic conditions but also by colonic cells during inflammation (32). Papadakis et al. have confirmed that the CCR9-CCL25 axis is associated with inflammation, particularly in small bowel CD (33). Interaction of CCL25 with CCR9 contributes to the homing of expressing α4β7+ T cells to intestinal sites (32). CCR9+ T cells isolated from patients with CD show significantly higher expression of IL-17 and IFN-γ after stimulation compared to healthy controls (34). In addition, expression of CXCR1 and CXCR2 is characteristic of neutrophils, which can bind to seven neutrophil attraction and chemotactic factors, CXCL1-3 and CXCL5-8, of which CXCL8 (IL-8) is the most efficient and abundantly secreted protein by the cells, inducing neutrophil infiltration from the blood into inflammatory tissues and amplifying inflammation (35). The involvement of neutrophils and CXCL8 in the pathogenesis and development of intestinal mucosal inflammation in IBD has been previously described (36).

### 2.2 Adhesion molecules in IBD

Lymphocyte homing is a process whereby lymphocytes in the blood migrate directionally and settle in peripheral immune organs or tissues, including lymphocyte recycling and lymphocyte migration to inflammatory sites (such as intestinal mucosa and skin). The molecular basis of lymphocyte homing is the interaction between lymphocyte homing receptors expressed on the surface of lymphocytes and adhesion molecules expressed on the surface of endothelial cells, wherein T lymphocyte trafficking is the most important (37, 38). Under the binding of chemokines to their cognate receptors on lymphocytes and other soluble pro-inflammatory cytokines, the expression of integrins (αLβ2 (lymphocyte function-associated antigen-1 (LFA-1), α4β1(very late antigen-4 (VLA-4), and α4β7) on the membranes of effector T cell subsets and cell adhesion molecules (intercellular adhesion molecule-1 (ICAM-1), vascular cell adhesion molecule-1 (VCAM-1), and MAdCAM-1) on endothelial cells are upregulated (15, 39). Firm interactions between cell adhesion molecules and integrins allow effector T cell subsets to adhere tightly to the vessel wall and eventually to transmigrate through the endothelial cells to the intestinal mucosal lamina propria (39). MAdCAM-1, a major ligand in effector lymphocytes that expresses integrin α4β7, is highly expressed in the venules of intestinal tissue in close contact with the intestinal epithelium (15), and its expression is strongly induced by TNF-α in patients with IBD (40). Since MAdCAM-1 is usually expressed only in gastrointestinal tissues, the interaction of MAdCAM-1 with α4β7 is thought to be exclusive to the migration occurring in the gastrointestinal tract (41). The interaction of αLβ2 with ICAM-1 and α4β1 with VCAM-1 contributes to the migration of intestinal effector lymphocytes (15). The expressions of ICAM-1, VCAM-1, and MAdCAM-1 are upregulated in the active site of intestinal inflammation in patients with IBD (16).

### 2.3 Th1 and Th17 pathways in IBD

Th1 and Th17 cells are crucial central pathways in the pathogenesis of IBD, and their mediators and upstream and downstream molecules are abundantly involved in the lesion (42). The most relevant cytokines that promote the differentiation of Th1 and Th17 cells are IL-12 and IL-23, respectively, which are mainly oversecreted by activated dendritic cells and macrophages (43, 44). IL-12 is a heterodimeric cytokine composed of IL-12p35 and IL-12p40 subunits that induces upregulation of the transcription factor, T-bet, in naïve T cells and promotes differentiation of Th1 cells that produce IFN-γ, TNF-α and recruit macrophages, natural killer cells, and CD8+T cells. In the presence of IL-1, IL-6, and transforming growth factor β (TGF-β), a cytokine, IL-23, composed of IL-12p40 and IL-23p19 subunits, can induce upregulation of the master transcription factor, RORγt, and promotes differentiation of Th17 cells, which produce IL-17, IL-22, and recruit neutrophils (1, 45). Innate lymphoid cells (ILCs) lie adjacent to the intestinal mucosal barrier and the effector cytokines secreted by them can modulate the pathology of IBD; for example, group 1 ILCs and group 3 ILCs produce IFN-γ, TNF-α, IL-17, and IL-22 (46). In the intestinal mucosa, IL-17 can induce the expression of pro-inflammatory cytokines (TNF-α, IL-1β, and IL-6), various chemokines (e.g., CCL20, which attracts Th17 cells, γδT cells, and dendritic cells; CXCL8/IL-8, CXCL1, which attracts neutrophils, and monocyte chemoattractant protein (MCP-1), which attracts monocytes and macrophages), and adhesion molecule (ICAM-1) (44, 47), resulting in profound pro-inflammatory effects. High levels of IFN-γ secreted by Th1 cells may activate vascular endothelial cells, leading to the infiltration of various immune cells from the blood into the intestinal mucosa (48). Simultaneously, IFN-γ itself, as the main product of Th1 cells, not only makes the immune response biased towards Th1 phenotype but also upregulates the expression of chemokines (such as CXCL9, CXCL10) that attract Th1 cells and MHC molecules on tissues cells and APCs (48). In chronic inflammation of the intestinal tract, the pleiotropic and strongly potent pro-inflammatory cytokine, TNF-α, can induce a wide range of pro-inflammatory effects, including activation of endothelial cells, expression of inflammatory chemokines, activation of macrophage and effector T cells, necrosis of Paneth cells, and damage to epithelial cells (49, 50). Presumably, IL-17 alone appears to elicit only a mild to moderate inflammatory cellular response but synergistically with IFN-γ and/or TNF-α enhances cellular IL-6, IL-8(CXCL8) synthesis and release, along with ICAM-1 expression (51, 52). A recent study showed that TNF-α and IFN-γ also act synergistically and lead to intestinal epithelial cell death through induction of the caspase-8-JAK1/2-STAT1 module (53). All of these indicate the role of these cytokines in the core network of IBD.

Although chemokines, adhesion molecules, and cytokines play an indispensable role in maintaining immune homeostasis and lymphoid tissue development, the occurrence of aberrant homing of lymphocytes in IBD indicates that the mechanisms are imperfect. Aberrant homing of autoreactive pathogenic T cells is closely related to many autoimmune diseases (54). For example, aberrant homing of gut-directed T lymphocytes in IBD may lead to autoimmune, chronic T lymphocyte-mediated cutaneous EIMs, characterized by specific effector mechanisms of different T lymphocyte subsets and non-specific inflammation.

## 3 Skin manifestations of IBD

### 3.1 Pyoderma gangrenosum

#### 3.1.1 Epidemiology

PG is the second most common cutaneous manifestation of IBD (55), occurring in approximately 0.5–2.6% of patients with IBD (56), and is more common in UC than in CD (57). Although PG can occur in all age groups and both males and females, it is more likely to occur in the elderly (~50 years old) and females (58, 59). Interestingly, up to 50% of patients with PG also have underlying IBD (60), which in most cases precedes the diagnosis of PG (7).

#### 3.1.2 Clinical characteristics

**Figure 2:**
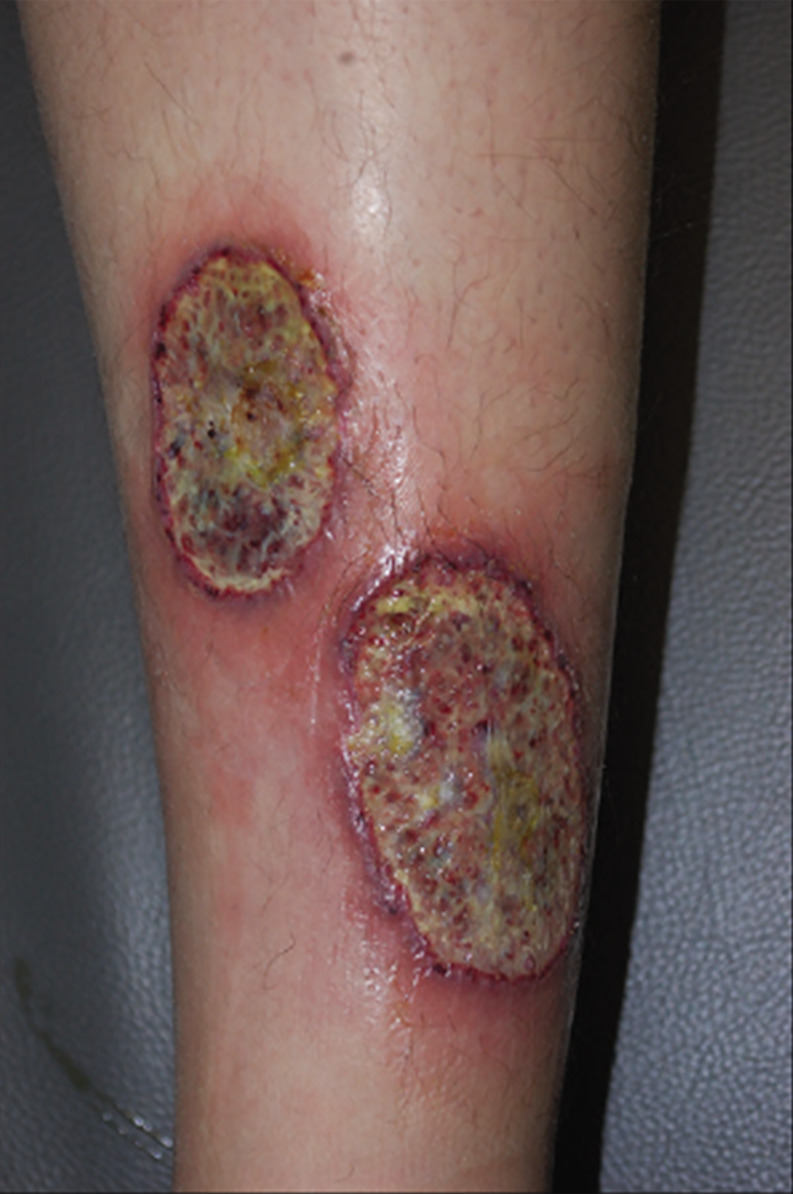
Ulcerative PG, the most common type, presents as a rapidly enlarged deep ulceration with erythematous borders. Reproduced with permission from (62). ^©^ 2023 Indian Journal of Dermatology, Venereology and Leprology - Published by Scientific Scholar.

PG is an immune-related autoinflammatory neutrophilic disease with severe and debilitating clinical behavior (57, 59). PG can be induced by minor trauma and usually presents as single or multiple tender inflammatory papules or pustules that rapidly break down over the next few days into painful necrotic ulcers ranging in size from 2–20 cm, with a characteristic violaceous undermined borders and peripheral erythema, accompanied by sterile purulent discharge (59, 61) (**Figure 2**). However, in severe cases, necrotic ulcers may form spontaneously even in the absence of a history of trauma (59). The base of a PG ulcer usually does not extend beyond the adipose tissue underlying the dermis and is initially an oozy exudative base, which can transform into exuberant granulation tissue within a few weeks (59). Although PG can occur in any part of the skin, extensor surfaces of the legs (shins) and adjacent to postsurgical stoma areas are most commonly involved (57). PG is often associated with several other immune-mediated diseases, most commonly IBD and rheumatoid arthritis (63).

#### 3.1.3 Histopathology

**Figure 3:**
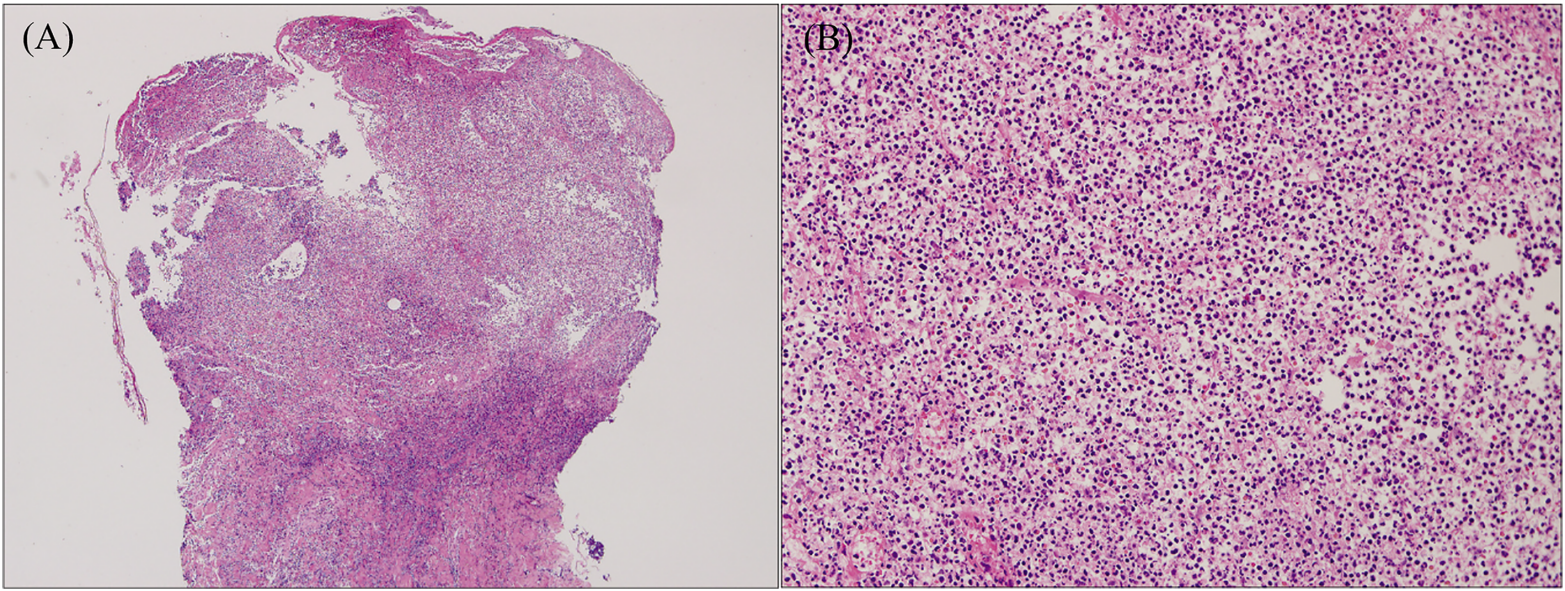
Histopathology of ulcerative PG. **(A)** Low-power magnification (H&E staining, ×125) reveals diffuse epidermal necrotic ulcer with massive neutrophilic infiltrations and dermal granulation tissue. **(B)** High-power magnification (H&E staining, ×2000) demonstrates that the majority of lesional cells consist of neutrophils. Reproduced with permission from (62). ^©^ 2023 Indian Journal of Dermatology, Venereology and Leprology - Published by Scientific Scholar.

The histopathologic features of PG are not specific (**Figure 3**). They can be explained by the chronology of lesions and one relies on the site of the biopsy (64). Early lesions present as deep perifollicular inflammation with a dense lymphocytic infiltration (64, 65). Ulceration of the epidermis is often secondary to inflammation of the dermis (64), and their centers show sterile purulent dermatitis and panniculitis with numerous infiltrations of macrophages and multinucleated histiocytes (66). Inflammatory cell infiltration, predominantly by lymphocytes, is often reported in the surrounding skin (64, 66). Biopsies of later-stage ulcers show a dense neutrophilic infiltrate with features of abscess formation (64). As the lesion regresses, plasma cells and macrophages intrude the dermis, and eventually, the inflammation is replaced by fibrosis (66).

#### 3.1.4 Immunopathophysiology

The pathogenesis of PG is not fully understood but is generally considered an autoinflammatory disease involving both innate and adaptive immune responses (59). Part of the mechanism may involve an adaptive immune response mediated by Th1 cells and Th17 cells (59). Histological analysis of early PG papules has revealed that perivascular and peripilosebaceous infiltrates are dominated by dense T lymphocytes rather than neutrophils (65). Gene expression analysis of these papules has revealed overexpression of genes encoding neutrophil-attracting chemokines (e.g., CXCL8/IL-8, CXCL3, CXCL5), Th1 cell-attracting chemokines (e.g., CXCL9, CXCL10, CXCL11), and multiple cytokines (e.g., IL-17, IFN-γ, TNF-α) (65). The genes of transcription factors consistent with the Th1 phenotype are up-regulated. Particularly, there is a strong up-regulation of the Th1 cell-promoting transcription factors, namely STAT1 (signal transducer and activator of transcription-1) and STAT4, along with the down-regulation of the Th2 cell-promoting transcription factor, GATA3 (65). There appears to be an imbalance between Th17 cells and regulatory T (Treg) cells in early PG, characterized by overexpression of Th17 cell-associated cytokines and a decrease in Treg cells (65, 67). These cytokines may be important factors driving the autoimmune inflammatory response of early PG. Th1 cell-mediated immune responses are important for the profound promotion and feedback amplification of inflammation, while Th17 cell-mediated immune responses are critical for the proliferation, maturation, and recruitment of neutrophils, which may be partially mediated by its upstream molecule, IL-23 (68–70). Moreover, neutrophils are the predominant cell type within the ulcer bed and below the destruction border in cases of advanced PG (59). Therefore, T cells in PG may be preferentially polarized into Th1 and Th17 cytokine secretion profiles, which are important sources of IFN-γ, TNF-α, and IL-17 (59), and play an important role in mediating organ specificity and tissue damage and the evolution of early-late-stage lesions in PG.

#### 3.1.5 Biologics that warrant consideration

The clinical behavior of PG is often debilitating and unpredictable. Despite treatment, it may persist. However, early and proactive intervention is necessary and attempts should be made to cure the lesion quickly. Since it is unclear whether PG occurs parallelly with the IBD activity (57), it is necessary to treat PG independently while treating IBD. Mild cases may be treated with local or topical treatments, including intralesional corticosteroid injections, moist dressings, and topical sodium cromoglycate 1-2% solution (59, 71) but systemic medications are usually required, including oral corticosteroids (prednisone 0.5–2 mg/kg/day, prednisolone 0.75 mg/kg/day), oral cyclosporine (3.5–5 mg/kg/day), and dapsone (50–200 mg/day) (59, 71, 72). Treatment with biologic agents has been reported to be effective in improving PG, most notably with the use of anti-TNF-α drugs. Only one randomized controlled trial for PG showed that the portion of patients reporting a clinical response was higher in the infliximab-treated (single dose of 5mg/kg) group compared to the placebo group (6/13 [46%] *vs* 1/17 [6%]) for the primary endpoint at week 2, and an overall complete remission rate (including 16 patients from the placebo group) of 21% (6/29) for the secondary endpoints of clinical remission at week 6 (73). A retrospective observational study by Argüelles-Arias et al. reported a cure rate of up to 92% (22/24) with infliximab and a healing time of 4–8 weeks (74), while a multicenter retrospective study by Regueiro et al. (single dose of 5mg/kg) showed cure rates as high as 100% (13/13) with complete healing times ranging from 1–30 weeks (75). Some case reports have shown that adalimumab (160/80 mg at 0 and 2 weeks, then 40 mg every other week) has a certain clinical effect on PG (76, 77). IL-12/23 antagonists may be a theoretically feasible therapeutic approach. Two recent retrospective multicenter studies have reported that ustekinumab is also clinically effective for treating patients with PG. Phillips et al. reported complete remission in all three patients with PG (median time to remission 4 months) (78), while De Risi-Pugliese et al. reported complete response in three and partial remission (> 50% reduction of symptoms) in one of four cases with PG (90 mg at 0, 4 and 8, and then every 8 weeks) at week 16 after initiating treatment (79). Although these studies suggest promising prospects for the clinical treatment of PG with biologics, large and randomized placebo-controlled clinical trials are needed to assess the efficacy and safety of these biologics for PG treatment.

### 3.2 Erythema nodosum

#### 3.2.1 Epidemiology

EN is the most common skin manifestation of IBD (55) and appears to be more prevalent in CD than in patients with UC (61). The prevalence of EN is 2–10% in patients with UC and 5–15% in those with CD (8). Although EN can occur in any age group, both in males and females, it appears to be more common in people between the ages of 20 and 30 years and females (12, 61, 80). Typically, EN is diagnosed later than IBD, and the proportion of patients with EN before IBD diagnosis is less than 15% (7).

#### 3.2.2 Clinical characteristics

**Figure 4:**
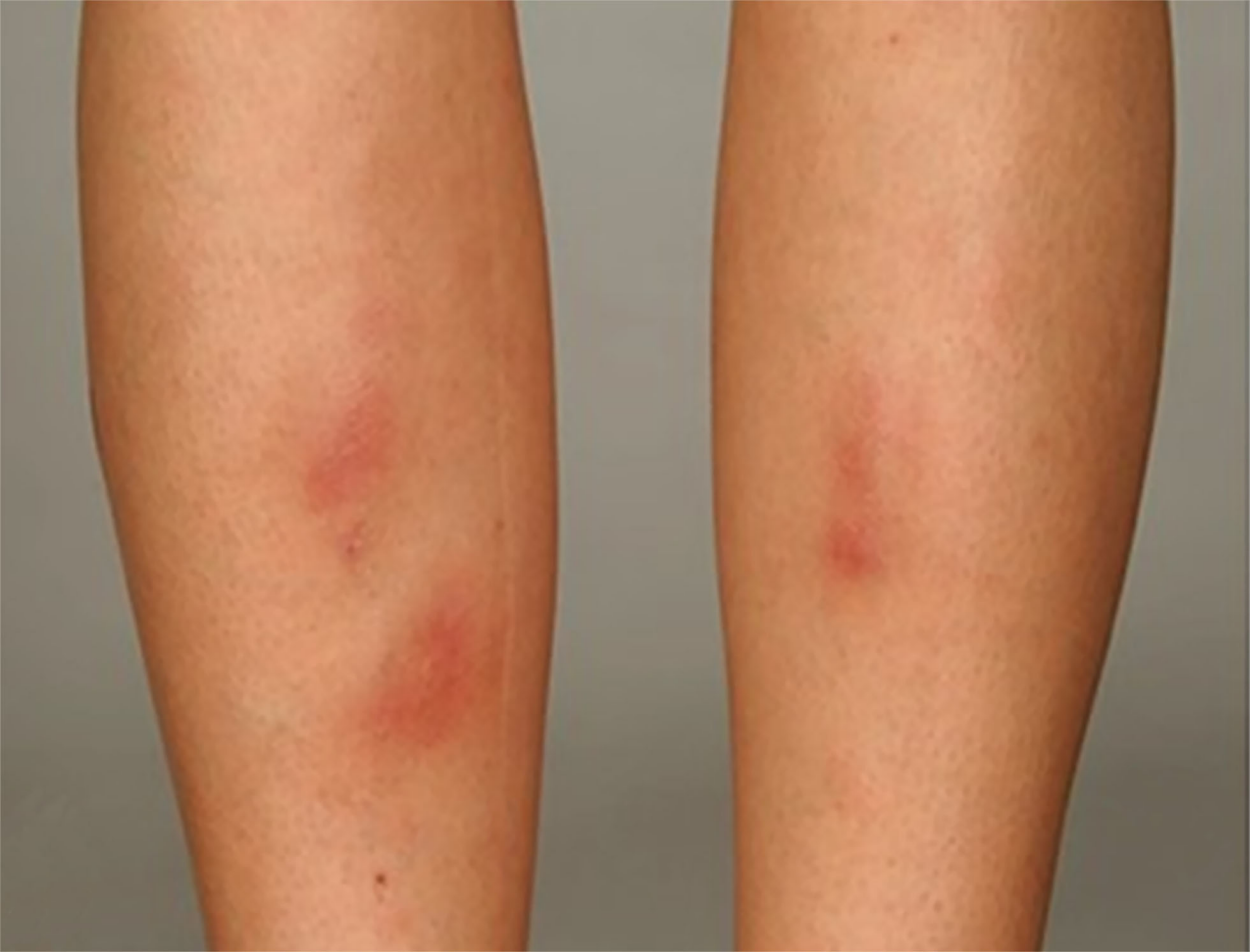
A typical EN shows symmetrical, raised, erythematous, tender non-ulcerative subcutaneous nodules on the tibial anterior region. Reproduced with permission from (57). ^©^ 2015 Crohn’s & Colitis Foundation of America, Inc.

EN is an immune-mediated inflammatory skin disease belonging to typical septal panniculitis and is associated with different pathological processes (81, 82). The main clinical features are symmetrical, raised, red, or violet tender non-ulcerative subcutaneous nodules 1–5 cm in diameter (81) (**Figure 4**). Typically, EN is self-limiting, with skin lesions mostly resolving within 2–8 weeks without ulceration, scarring, or atrophy (82, 83). EN is usually symmetrically distributed over the extensor surface of the lower extremities, most commonly in the anterior tibial region, and rarely affects the face, trunk, and upper extremities (57). EN is not only associated with IBD but is seen in other immune/non-immune mediated diseases, including Behçet’s syndrome, systemic lupus erythematosus, sarcoidosis, infection, and on some medications (82, 83).

#### 3.2.3 Histopathology

**Figure 5:**
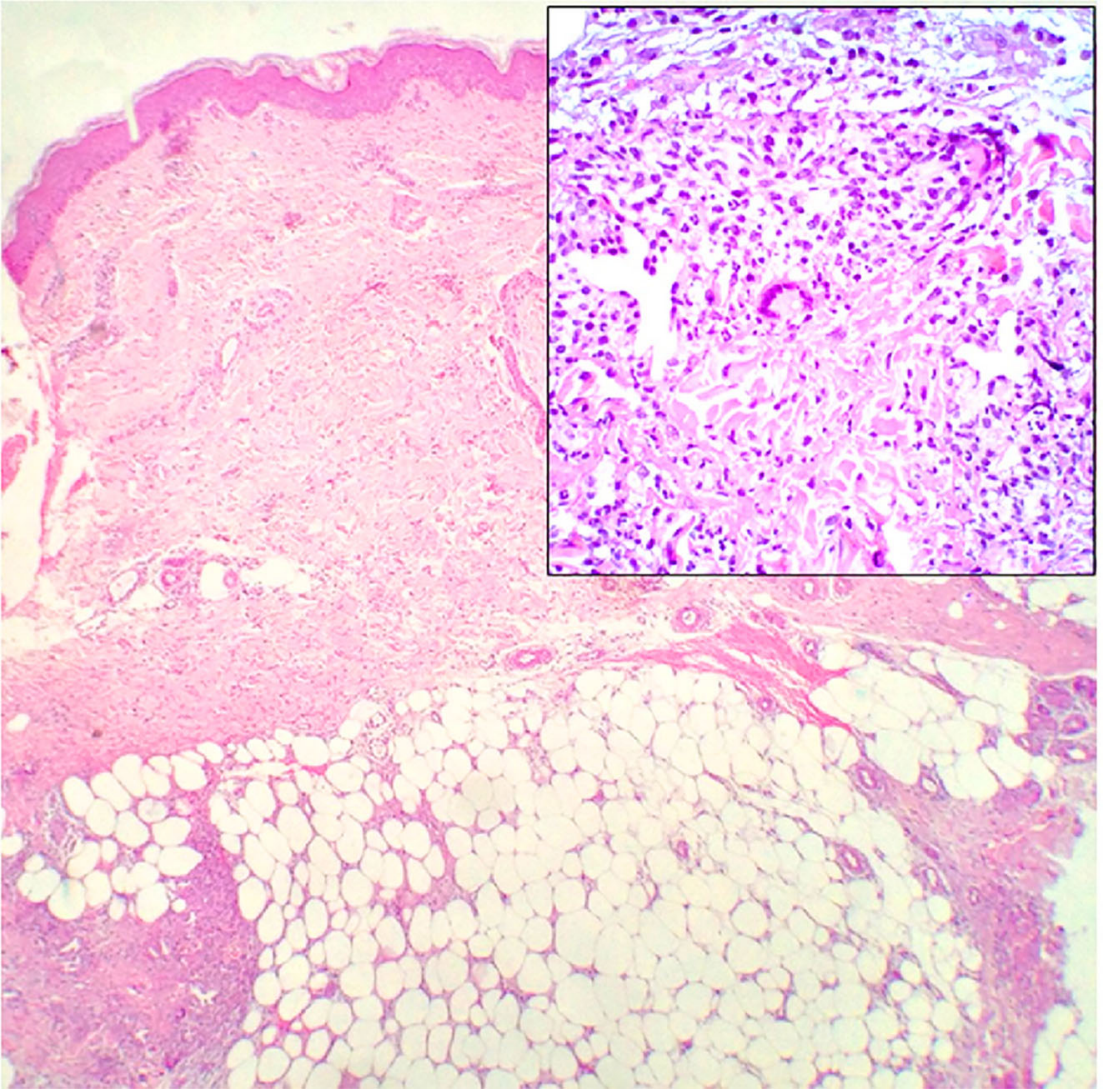
Histopathology of EN shows septal panniculitis and a mixed inflammatory infiltrate predominantly composed of lymphocytes, histiocytes, and neutrophils (H&E staining, ×40). Inset: Miescher’s radial granuloma (H&E staining, ×200). Reproduced with permission from (84). ^©^ 2016 The International Society of Dermatology.

Although EN is associated with the pathological processes of a variety of diseases, histopathological examination of the lesion site demonstrates typical septal panniculitis without vasculitis, regardless of the underlying etiology (83, 84). It usually changes with the phase of the disease (85). The early lesions show interlobular septal edema with mixed inflammatory cell infiltration, including lymphocytes, histiocytes, eosinophils, and numerous neutrophils (**Figure 5**). Inflammation is usually concentrated in the periphery of the septa. The late lesions present thickening and fibrosis of the septa infiltrated by lymphocytes, histiocytes, multinucleated giant cells, and a few neutrophils. Inflammation extends to the periphery of the fat lobules (82, 85, 86). Miescher’s radial granulomas can also be observed (84) (**Figure 5**). Miescher’s radial granulomas present with nodular aggregations of small histiocytes and macrophages radially arranged around star-shaped cleft-like spaces, which are typical characteristics of EN (82, 83, 86).

#### 3.2.4 Immunopathophysiology

The underlying pathogenesis of EN is complex and not fully understood, but part of the pathogenesis of EN is considered to involve type IV hypersensitivity reaction (71). Histopathology of the lesion site has revealed abundant infiltration of lymphocytes, histiocytes, and neutrophils in the lower dermis (86), with neutrophils usually predominating in the early lesions (55). Furthermore, a previous study found significant overexpression of genes for encoding Th1 cytokines (IL-2, IFN-γ) in cutaneous lesions and peripheral blood of patients with EN (87). Correspondingly, Simone et al. in 2016 also found elevated levels of Th1 cytokines in the lesional skin and serum of patients with EN with particularly high levels of IFN-γ and IL-12 (84). These findings provide direct evidence that a polarized Th1 immune response is present in the skin lesions of patients with EN. Simultaneously, high levels of cytokines mainly involved in neutrophil recruitment and activation, including IL-6, IL-8, TNF-α, granulocyte colony-stimulating factor, and MCP-1, are present in the skin of the lesion site and serum (84). However, Simone et al. found that IL-17 levels were low in both serum and skin samples in five patients, and that the balance between Th17 cells and Treg cells varied widely in the five enrolled patients. Therefore, they believe that IL-17 and Th17 cells play a negligible role in the pathogenesis of EN (84). Although IL-12 can induce IFN-γ production by Th1 cells, thereby enhancing the expression of chemokines to promote neutrophil recruitment and activation (88), IL-17 is also a powerful pro-neutrophil cytokine, critical for neutrophil proliferation, maturation, and chemotaxis (68). Given the primary EN, small sample size, and lack of healthy skin controls included in this study, the role of IL-17 and Th17 cells in EN secondary to IBD may be controversial. Shimizu et al. found significantly higher expression of IL-17 and TGF-β in EN secondary to Behçet’s syndrome compared to patients with primary EN (89). Moreover, another study found that STAT3, which is required for up-regulation of the master transcription factor RORγt, is not only overexpressed in IBD and PG but also significantly overexpressed in EN (90). Therefore, IL-17 and Th17 cells may be upregulated in EN secondary to IBD. Many adhesion molecules and inflammatory mediators are also involved in the development of EN. The expression of several adhesion molecules has been observed on the endothelial cells of patients with EN, such as E-selectin, P-selectin, platelet endothelial cell adhesion molecule-1, VCAM-1, and ICAM-1 (91). The formation of circulating immune complexes and deposition in and around venules of the connective tissue septa of the subcutaneous fat suggest that antigens and antibodies may also play an important role in the pathogenesis of EN (92, 93).

#### 3.2.5 Biologics that warrant consideration

In cases of EN secondary to IBD, as the skin lesions reflect the activity and onset of intestinal disease, treatment of the underlying IBD usually results in remission of the skin manifestations (55). EN is usually self-limiting, even without specific treatment for the cause, and symptomatic supportive treatment including compression bandages, limb elevation (> 30 min twice daily), analgesics, and potassium iodide (300 mg three times daily, 2-3 weeks after remission) is typically sufficient for most patients (57, 71, 85). For severe or refractory cases, systemic corticosteroids (prednisone 20 mg for 7-10 days) may be considered as the first-line therapy (57, 61, 71). Anti-TNF treatment may serve as rescue therapy, especially in patients with active IBD. An analysis from the Swiss IBD Cohort Study showed the best response rate of up to 80% (8/10) with anti-TNF-α treatment for EN (94). A multicenter, open-label, Phase 3b study by Löfberg et al. showed that adalimumab (160/80 mg at 0 and 2 weeks, then 40 mg every other week) reduced the incidence of EN from 2.4% (23/945) at baseline to 0.4% (4/942) on the clinical endpoints at 20 weeks (95). Additionally, several case reports have highlighted the benefits of adalimumab (40 mg every 14 days) and infliximab (5 mg/kg at 0, 2, and 6 weeks) in the treatment of severe or refractory EN (96, 97). Recent studies have found that monoclonal antibodies against the IL-12/23p40 subunit may exert some clinical effects for the treatment of EN. A multicenter case series by Phillips et al. reported four complete remissions and one partial remission in five patients with EN treated with ustekinumab (78). A prospective multicenter study of 221 patients with CD (the initial intravenous infusion with ustekinumab based on weight [260 mg < 55 kg, 390 mg between 55 and 85 kg, 520 mg > 85 kg], 90 mg subcutaneously at 8 weeks, then 90 mg every 8 or 12 weeks) reported data on EN. Two patients had EN at baseline and 50% (1/2) of patients presented with the primary outcome of corticosteroid-free clinical remission. Three patients developed new-onset EN during follow-up, with a response rate of 66.7% (2/3) (98).

## 4 Potential pathophysiological associations between IBD and cutaneous EIMs

The underlying pathophysiological mechanisms of IBD and cutaneous EIMs are complex, diverse, and not fully understood. EIMs reportedly occur at a frequency of 6–47% in the lifetime of patients with IBD, with the skin being among the most commonly affected organs (7). There may be a common pathogenic association between IBD and cutaneous EIMs. Further, two different theories have been proposed to explain this potential pathogenic association. One is that EIMs are only independent inflammatory events that share a genetic background or environmental risk factors with IBD, and the other is that EIMs may be the result of an extraintestinal extension of intestinal inflammation (99). These mechanisms are not mutually exclusive and often multiple pathological mechanisms may interact to promote the occurrence and progression of skin EIMs. Understanding the potential association between IBD and cutaneous EIMs is critical for identifying targeted treatment strategies in the future.

### 4.1 Aberrant lymphocyte homing

Aberrant homing of gut-specific lymphocytes in IBD may contribute to extraintestinal skin manifestations of IBD (100). MAdCAM-1 is typically expressed exclusively in the gut, and its interaction with the α4β7 integrin selectively transports gut-tropic T cells to the intestinal mucosa (41). The interaction of the gut-expressed chemokine, CCL25, with its corresponding chemokine receptor, CCR9, also contributes to lymphocyte homing to the gut site (32). Thus, the ectopic expression of gut-specific adhesion molecules and chemokines may facilitate the transport of inflammatory T lymphocytes to extraintestinal sites. In PSC, ectopic expression of MAdCAM-1 and CCL25 on the vascular endothelium of the portal tract has been demonstrated (101, 102). However, apart from PSC, evidence that this condition can occur in organs or tissues other than the liver, especially in the skin, is scarce. Vavricka et al. analyzed 31 biopsy samples from EN and PG and showed that gut-specific MAdCAM-1 was not overexpressed in the skin (90). Despite the lack of gut-specific expression of MAdCAM-1 in the skin, a recent large real-life experience cohort study found that vedolizumab (an anti-human α4β7 integrin antibody) resulted in complete remission rates of up to 75% in the treatment of cutaneous EIMs (103). It is unclear whether this is a direct effect of vedolizumab on cutaneous EIMs or an indirect effect through the control of intestinal disease activity. Lymphocytes may also co-express gut-specific and skin-specific homing molecules, which may allow the tropism of lymphocytes in IBD toward skin sites (104). For example, Th17 cells can simultaneously express CCR4 and CCR6, which can mediate Th lymphocyte chemotaxis to skin sites and intestinal lymphoid tissues, respectively (27, 31). γδT cells can co-express integrin α4β7 and skin-homing molecules like cutaneous lymphocyte-associated antigen (CLA) (105) (**Figure 1**).

The up-regulation of non-gut-specific chemokines and adhesion molecules at inflammatory sites may also mediate the transport of T lymphocytes to extraintestinal sites. Both intestinal epithelial cells and skin keratinocytes secrete CCL20, a chemokine strongly upregulated by inflammatory stimulation, which attracts the recruitment of Th17 cells expressing its cognate receptor, CCR6 (27, 106) (**Figure 1**). CCL27 expressed by skin keratinocytes and CCL28 expressed by intestinal mucosal epithelial cells have high homology, and both chemotactically recruit T lymphocytes expressing CCR10 (**Figure 1**). Thus, inflammatory T lymphocytes derived from the gut and expressing CCR10 may be recruited to skin sites (107). Simultaneously, non-specific chemokines CXCL9, CXCL10, and CXCL11 are produced at the site of inflammation, which may attract Th1 cells and γδT cells from the intestinal site of patients with IBD expressing the receptor CXCR3 to the skin site (21, 105) (**Figure 1**). In addition, non-specific ligand and receptor interactions between lymphocytes and endothelial cells may also contribute to the transport of lymphocytes to extraintestinal sites. Human circulating γδT cells express LFA-1 (αLβ2), which primarily recognizes ICAM-1 expressed by endothelial cells and which further increases under inflammatory conditions and tissue stress (105) (**Figure 1**). Thus, in the context of a genetic predisposition of patients with IBD, low-grade inflammation, injury or mechanical stress of skin against external hazards, toxic substances, and pathogens may lead to increased concentrations and recruitment of Th1, Th17, and γδT cells in the gut at the diseased skin (99). In conclusion, specific and non-specific lymphocyte-endothelial cell interactions may induce ectopic homing of intestinal lymphocytes to skin sites, leading to the development of cutaneous EIMs.

### 4.2 Th1 and Th17 pathways and the TNF pathway

Th1 and Th17 pathways and the TNF pathway play an important role in the occurrence and development of IBD. In IBD, exposure to pathogenic microorganisms, antigen presentation by APCs, and interaction of APCs with Th cells lead to macrophage activation. Activated macrophages produce cytokines with profound pro-inflammatory effects, including TNF-α, IL-12, and IL-23 (1). TNF-α binds to death receptors of the TNF receptor superfamily resulting in intracellular signaling (108), while IL-12 and IL-23 act by binding to IL-12 receptors (consisting of IL-12Rβ1 and IL-12Rβ2) and IL-23R (consisting of IL-12Rβ1 and a novel IL-23R), respectively (109, 110). The p40 subunit shared by IL-12/23 binds to the transmembrane IL-12Rβ1 on the cell surface (109, 111). A monoclonal antibody (ustekinumab) directed against the p40 subunit shared by IL-12 and IL-23, as well as a monoclonal antibody against TNF-α, are effective in the treatment of CD and UC (112–114). Therefore, inhibition of Th1 and Th17 signaling pathways and the TNF-α pathway has become a successful strategy for the treatment of IBD. Recent studies have shown that anti-TNF-α is effective in the treatment of cutaneous EIMs (94, 115), and ustekinumab is also effective in the control of cutaneous EIMs (116). An analysis based on the Swiss IBD Cohort Study showed that response rates to anti-TNF-α therapy were 80% (8/10) for EN and 50% (6/12) for PG (94). A prospective multicenter study of 221 patients with CD treated with ustekinumab reported data on EN. Two patients had EN at baseline and 50% of patients showed clinical remission. Three patients developed new EN during follow-up, with a response rate of 66.7% (98). Another retrospective multicenter study evaluated the efficacy of ustekinumab in neutrophilic dermatosis associated with CD. Four of the seven CD patients included had PG, with three complete healings and one partial response defined as more than 50% improvement of the skin lesions (79). These data suggest a common Th1 and Th17 pathway- and TNF-α pathway- based pathogenic association between IBD and cutaneous EIMs, and between different cutaneous EIMs (**Figure 1**). In fact, in early PG studies a large number of Th1 and Th17 cell infiltrates as well as high levels of TNF-α, IFN-γ, IL-12, IL-17, and IL-23 at the diseased skin have been reported (59). These cytokine profiles are similar to the results observed in PG-associated diseases such as IBD (1), suggesting that the Th1 and Th17 pathways, as well as the TNF-α pathway, underlie commonly, associating together these diseases. There is growing evidence that the Th1 and Th17 pathways, as well as the TNF-α pathway, are involved in the pathogenesis of cutaneous EIMs. However, it is unclear whether this is the result of an extension of intestinal disease activity to the skin or a widespread pro-inflammatory cytokine imbalance in genetically predisposed patients. However, the detection of high levels of TNF-α, Th1 cytokines, and Th17 cytokines in the sera of patients with IBD seems to confirm the latter possibility. These high levels of pro-inflammatory cytokines associated with IBD may lead to the occurrence of cutaneous EIMs by activating immune cells at the skin site.

### 4.3 Antigen cross-reactivity and/or similarity with microbial antigens

Both the gut and skin are active, complex immune and neuroendocrine organs that are constantly exposed to the external environment and have a broad microbial community (117, 118). Many studies have associated gastrointestinal health with skin homeostasis and dysregulation and have proposed the concept of a gut-skin axis. There is evidence of a bi-directional interaction between the gut and the skin, which is mainly achieved through immuno-crosslinking (118, 119). Polkowska-Pruszynska et al. discussed the role of dysregulation of the gut microbiota in the pathogenesis of a range of autoimmune inflammatory skin diseases, including psoriasis, hidradenitis suppurativa, atopic dermatitis, and alopecia areata (119). Intestinal dysbacteriosis is associated with some less common but more serious skin diseases, such as EN and PG (120). Although specific skin disorders are associated with gut health and the balance of the microbiome within the gut, the exact mechanisms by which gut microbes affect the skin have not been fully elucidated. The cross-reactivity of antigens (molecular mimicry) plays an essential role in the pathogenesis of autoimmune diseases (121). Autoimmune reactions can occur when foreign antigens (from bacteria or viruses) have sequence and/or structural similarities to self-antigens (121). Molecular mimicry has typically been characterized for T cells and at the antibody level (121). Antigens from the gut microbiota are thought to be key targets for gut effector T cells (119). Similar pathogenic microbial communities between the gut and skin may exist (99) which produce similar microbial antigens. Thus, in the context of IBD, cells at the skin site may be reactivated by similar antigens or host cross-antigens of the gut and skin microbiota, thereby presenting skin EIMs. Mice with reduced or absent gut microbiota have significantly lower levels of γδT cells, Th17 cells, and Th1 cells both locally and systemically, and imiquimod can hardly induce the development of skin inflammation (122). Gut microbes drive psoriasis-like skin inflammation in mice by inducing stronger γδT cell, Th17 cell, and Th1 cell responses (122), which are likely to be cross-reactive bacterial antigens, providing evidence for a direct association between gut microbiota, cross-reactive autoantigens, and skin inflammation. Presently, whether modulating the gut microbiota can effectively target the treatment of cutaneous EIMs is uncertain. Satta et al. have shown that the use of probiotics is significantly inversely associated with the occurrence of skin lesions in patients with IBD (123).

### 4.4 Circulating autoantibodies

Circulating autoantibodies can extend the intestinal immune inflammatory response to extraintestinal sites, and immune complex-mediated inflammation can contribute to cutaneous EIMs (124). Earlier studies have demonstrated the presence of autoantibodies which are reactive to colonic proteins in patients with IBD (125, 126), and share certain unique epitopes between the human colon and skin, as evidenced by the presence of monoclonal antibodies (127). However, the exact role of these autoantibodies or immune complexes in promoting the development of cutaneous EIMs in patients with IBD is unclear.

### 4.5 Abnormal neutrophil functions

Neutrophils are the major circulating white blood cells in the human body. In healthy individuals, circulating neutrophils are in the “dormant state”. When neutrophils are exposed to infection and with subsequent release of inflammatory cytokines or other signals, these are primed, activated, and recruited to the site of infection, producing and releasing large amounts of antibacterial peptides, proteases, and reactive oxygen species (ROS), all of which contribute to host defense. Physiologically primed and activated neutrophils can be deprimed and deactivated and can return to circulation as quiescent neutrophils or eventually die by apoptosis. However, in inflammatory diseases, neutrophils can be highly primed and persistently activated, leading to tissue damage and excessive inflammatory responses (128). Circulating neutrophils in patients with IBD present morphological evidence of activation (129) and secrete higher levels of TNF-α and IL-1β (130). Primed neutrophils may fail to deprimed and then re-enter the bloodstream in a primed state (128) (**Figure 1**). Thus, in IBD, these neutrophils activated in the gut may be re-activated in the skin, resulting in skin damage and inflammation. The two most common cutaneous EIMs in IBD, PG and EN, present a large amount of neutrophil infiltration at the lesion site (55, 59). Magro et al. showed clonal neutrophil infiltration that is not associated with myeloproliferative disease in both PG and Sweet’s syndrome (131). A recent study by Jatana et al. demonstrated inflammatory skin-gut crosstalk is mediated by IL-1β-primed neutrophils in a murine model of PG with intestinal inflammation (132).

### 4.6 Other relevant pathological associations

In addition to the above mechanisms, some pathological associations exist. In IBD, systemic inflammatory cytokines, leakage of microbial products into circulation due to increased intestinal permeability, or changes in metabolites of the intestinal microbiota can all lead to altered innate immunity (99). For example, the impaired pro-inflammatory cytokine secretion by macrophages in patients with CD results in a reduced recruitment of neutrophils to the skin, causing reduced or delayed bacterial clearance from the skin site (133, 134), and ultimately, resulting in inflammation or damage at the skin site. Short-chain fatty acids (SCFAs) produced by gut commensal microbes facilitate the generation of peripheral anti-inflammatory Treg cells (135). Several studies have confirmed that compared to healthy subjects, the abundance of SCFA-producing bacteria in the feces of patients with IBD decreases significantly (136, 137), further inhibiting the generation of peripheral Treg cells and causing the disruption of the balance between pro- and anti-inflammatory in the skin. Additionally, intestinal inflammation promotes the differentiation of fibroblast subsets in the intestinal stroma into preadipocytes with innate antimicrobial activity (138). In IBD, preadipocytes mature into “creeping fat” with independent pro-inflammatory functions, and this process depends on the presence of hyaluronan (HA) in the extracellular matrix (138, 139). Skin inflammation degrades the matrix composed of extracellular HA. The degraded HA fragments enter the intestine through the circulatory system, thereby exacerbating this reactive fat maturation process. Therefore, skin inflammation exacerbates intestinal inflammation through HA (138).

## 5 Conclusions

In summary, extraintestinal skin manifestations of IBD are common and its manifestations range from mild and self-limiting (e.g., EN) to severe and debilitating courses (e.g., PG). The presence of cutaneous EIMs significantly affects morbidity and mortality due to IBD. Control of intestinal inflammation is effective for cutaneous EIMs associated with intestinal disease activity (e.g., EN), and multiple biologic agents are efficacious in treating cutaneous EIMs that develop independently of intestinal disease activity (e.g., PG). Clinicians may consider this treatment option to manage cutaneous EIMs. However, prospective and controlled studies with larger sample sizes are needed to confirm the efficacy and safety of various biologics in cutaneous EIMs. Common genetic backgrounds and risk factors are increasingly being recognized. Although some parts of the pathogenic mechanisms remain controversial, they are not mutually exclusive and may contribute to different skin EIMs to different degrees and be the result of multiple pathological mechanisms acting together. Therefore, elucidating the potential pathological associations between IBD and cutaneous EIMs is expected to help identify more new therapeutic targets in the future.

## Author contributions

RH wrote the first draft of the manuscript. RH and SZ prepared the figures and table. RH, SZ, and XW conceived the central idea and finalized the paper. SZ, MC, YC, JM, and JL prepared the material and collected the data. XW revised the manuscript critically for intellectual content and supported the funding. All authors contributed to the article and approved the submitted version.
